# Sociodemographic and Behavioral Risk Correlates of PrEP Interest and Use Among Young Adults Experiencing Homelessness in Los Angeles

**DOI:** 10.1007/s10461-023-04144-7

**Published:** 2023-09-12

**Authors:** Erik D. Storholm, David J. Klein, Eric R. Pedersen, Elizabeth J. D’Amico, Anthony Rodriguez, Rick Garvey, Joan S. Tucker

**Affiliations:** 1 School of Public Health, San Diego State University, San Diego, CA United States of America; 2 RAND Corporation, Santa Monica, CA United States of America; 3 Department of Psychiatry, Keck School of Medicine, University of Southern California, Los Angeles, CA United States of America; 4 RAND Corporation, Boston, MA United States of America

**Keywords:** Homeless, Young Adults, PrEP, HIV-prevention, Sexual Risk, Substance Use

## Abstract

Young adults experiencing homelessness (YAEH) are at elevated risk for HIV compared to their stably housed peers. Preexposure prophylaxis (PrEP) is highly effective at preventing HIV infection, yet YAEH have been largely overlooked in PrEP efforts to date despite YAEH reporting high overall interest in PrEP. We assessed individual, social, and structural variables associated with PrEP interest and use among a sample of 195 YAEH (ages 18–25) recruited from drop-in centers across Los Angeles County who met criteria for HIV risk. In the current sample, though most had heard of PrEP (81.0%), the majority were not interested in taking PrEP (68.2%) and only a minority had used/were using PrEP (11.8%). YAEH who identified as sexual and/or gender minority, reported knowing someone who had used PrEP, or recently accessed sexual health services were more likely to have used and/or reported interest in using PrEP. Those who reported more episodes of heavy drinking were less likely to report having used PrEP. Suggestions are provided for better integrating PrEP-related services into existing behavioral and health service programs for YAEH, as well as leveraging peers and fostering positive social norms to reduce PrEP-related stigma and increase interest and use of PrEP among YAEH.

## Introduction

Nearly 10% of young adults ages 18 to 25 experience homelessness each year in the United States [1]. Compared to housed youth populations, young adults experiencing homelessness (YAEH) self-report engaging in HIV risk behaviors (e.g., inconsistent condom use; having multiple or concurrent sexual partners; trading sex) more frequently [2–4] Approximately 8% of YAEH report HIV infection [5] and YAEH are 16 times more likely to be diagnosed with HIV than their stably housed peers [6]. YAEH are also among the marginalized populations at highest risk for mental health problems [7, 8] and substance use, [7, 9] both of which have been independently associated with HIV transmission risk among YAEH [10–13].

Preexposure prophylaxis (PrEP) is a highly effective biomedical intervention for preventing HIV infection [14–21]. PrEP is currently widely promoted as a prevention strategy for individuals at risk for HIV. Despite PrEP being indicated for YAEH, [22] and high reported interest in taking PrEP among YAEH, [23] prior work shows that a disproportionately low number of YAEH have been reached by PrEP interventions to date [24, 25]. This may be due, in part, to ongoing concerns about the ability of YAEH to consistently take daily PrEP pills in order to remain protected [26]. With the recent FDA approval of a long-acting injectable formulation of PrEP (lasting 1–2 months), there is now an additional option available for YAEH who may otherwise struggle with adherence to daily pill regimines [27].

Previous work has indicated significant individual, social, and structural barriers to PrEP uptake for young adults in general. Individual factors have included low levels of PrEP knowledge, [28] perceived high cost, [28, 29] mental health factors like depression, [30, 31] and substance use [30, 32]. Social factors have included experiencing PrEP-related stigma [33] and lack of social support from peers for PrEP [33, 34]. Structural factors have included lack of insurance coverage, transportation difficulties, housing instability [32] and lack of access, [29] engagement, and retention in health care [32]. Although knowledge of PrEP is relatively low among YAEH, a majority report willingness to take PrEP once informed about it. For example in a study of over 1,400 YAEH across seven US cities, only 29% had ever heard about PrEP but once informed about it, 59% reported they were likely to take it if available [25]. This highlights an important missed opportunity to inform and offer PrEP to YAEH. Currently little is known about factors associated with interest and use of PrEP among YAEH.

The current study addresses these gaps in the literature through two aims. The first aim assesses overall levels of awareness, interest, and use of PrEP among YAEH. The second aim identifies individual, social, and structural variables associated with PrEP interest and use. Participants in the current study comprise a diverse sample of YAEH ages 18–25 accessing services from drop-in centers serving youth experiencing homelessness in Los Angeles County.

## Methods

### Participants

Participants were a subsample of individuals participating in a cluster crossover randomized controlled trial evaluating a substance use and sexual health risk reduction program for 18- to 25 year olds experiencing homelessness [35]. Individuals were recruited to participate in the trial when accessing services (e.g., food, clothing) from one of three youth drop-in centers in Los Angeles County and were compensated for their participation. Of the 276 participants in the trial, they were eligible for the present analyses if they met one or more of the following HIV risk criteria: identifying as transgender, gender nonbinary, or as a cisgender sexual minority man who has sex with men; report having received an STI diagnosis, HIV test, or accessing HIV/STI services in the last 3 months; report engaging in condomless sex in the last 3 months; or report use of illicit substances other than cannabis (e.g., methamphetamine, heroin) in the past 30 days. Furthermore, participants must have completed the 6-month follow-up survey and responded to a question (described below) regarding PrEP interest or use. This resulted in an analytic sample of N = 195. All study protocols and measures were approved by the Human Subjects Protection Committee of the RAND Corporation. Further details on recruitment strategy and study design are available in the published protocol [35].

### Measures

A scannable self-report paper-pencil baseline survey was administered in English to participants who provided written consent to participate in the study. A staff member was present to provide assistance when completing the survey. A 6-month follow-up survey was conducted either in-person, via web-based administration, or by phone. The current study utilized measures of sociodemographic, mental health, substance use, sexual behavior, HIV/STI testing, diagnosis, and service access from the baseline survey and PrEP awareness, interest, intent, and current or past PrEP use from the 6-month follow up survey when the PrEP items were added.

#### Socio-Demographic Characteristics

Items included age, gender identity, sexual orientation, race and ethnicity, age of first homeless episode, length of current homeless episode, education, and employment status. To reduce multicollinearity for analytic purposes, we collapsed sexual and gender minority status into a 3-level variable consisting of cisgender females combined with straight cisgender males, gay and bisexual cisgender men, and gender diverse individuals.

#### Mental Health

Mental functioning was assessed with the eight-item Patient Health Questionnaire (PHQ-8) for symptoms of depression in the past 2 weeks (e.g., feeling down, depressed, or hopeless; 0 = *not at all* to 3 = *nearly every day*) [36]. The resulting score had a range of 0–24, and participants were classified as depressed if their score was 10 or greater [36].

#### Substance Use

Items from the Monitoring the Future survey [37] assessed number of days, in the last 30 days that participants engaged in substance use, including heavy alcohol use (5 + drinks in one day), cannabis use, and use of other illicit drugs (e.g., heroin, methamphetamine, hallucinogens).

#### Sexual Behavior

Participants completed separate items asking about the number of times they had anal or vaginal sex with primary partners and with casual partners, as well as the number of these times that they used a condom, in the last 3 months. Based on this information we derived variables indicating the percentage of sexual events with primary partners and with casual partners that were condom-protected (0-100%).

#### HIV/STI Testing/Diagnosis and Service Access

Participants reported if they had been tested for HIV in the last 3 months, as well as whether they had been diagnosed with a sexually transmitted infection (STI; including, but not limited to HIV) during this period. Separate items assessed whether participants had accessed formal mental health, sexual health, substance use, or housing services during the past 90 days.

#### Social Support from Friends

Participants were asked to rate the degree to which they received support from their friends during the last 30 days with 3 items from the PROMIS Pediatric Peer Relationships Scale (e.g., *I was able to count on my friends, my friends and I helped each other out*) [38]. Participants answered on a 5-point Likert-type scale with answers ranging from 1 = *never* to 5 = *almost always*.

#### PrEP Awareness, Interest, Intent, and Current or Past Use

To assess knowledge of PrEP, participants were asked two items of whether they had heard of PrEP (yes/no) and whether they knew anyone who had used PrEP (yes/no). To assess experience with PrEP, participants were asked to select one of the following responses: currently using PrEP; had used PrEP in the past, but were not currently using it; were interested in using PrEP and had already made plans to use it; were interested in using PrEP, but had not made any plans to use it; or had no interest in using PrEP. PrEP items were preceded by a definition of PrEP as “where someone who does not have HIV takes HIV medications to reduce the chances of being infected (before being possibly exposed to HIV).” For the purposes of analyses, we categorized participants into 3 groups: (a) Current/prior PrEP use; (b) PrEP interested but no prior use; and (c) not interested in PrEP and no prior use.

### Data Analysis

Bivariate multinomial logistic regressions were used to examine associations between each predictor variable of interest and the likelihood of: (1) current/prior PrEP use; or (2) PrEP interested but no prior use, with not interested in PrEP and no prior use serving as the reference. A multivariable multinomial logistic regression model was then evaluated in which all marginally significant (p < 0.10) bivariate predicators of PrEP current/past use and/or PrEP interest were included as independent variables. All analyses were conducted using SAS statistical software v9.4 [39].

## Results

### Participant Characteristics

The analytic sample consisted of 195 YAEH who reported not being HIV-positive and who met one or more HIV risk inclusion criteria. Table 1 presents sample characteristics. The majority of participants (80.5%) were between 21 and 25 years of age, with about 20% between 18 and 21 years of age. The sample was highly diverse in terms of racial and ethnic backgrounds with 37.1% identifying as non-Hispanic Black or African American, 30.4% identifying as Hispanic/Latinx, 16.5% identifying as non-Hispanic White, and the remaining 16.0% identifying as another non-Hispanic racial category or multiracial. The majority of the sample (61.5%) identified as cisgender male, 23.1% identified as cisgender female, and 15.4% identified as gender diverse. Half of the sample (51.0%) identified as straight/heterosexual, 18.8% identified as gay/lesbian, and the remaining 30.3% identified as bisexual/other. Just under half (45.3%) experienced homelessness for the first time prior to age 18. There was wide variation in the length of most recent homeless episode with answers ranging from less than a month to over two years. There was also wide variation in terms of education level with just under one third (32.0%) having not finished high school, 42.3% having obtained a high school diploma or equivalent, and about one-forth (25.8%) having more than a high school degree. The majority of our sample (76.8%) reported being currently unemployed.

**Table 1:** Characteristics of participants (N = 195)

Variable	%
**Age**	
18–20	19.5
21–25	80.5
**Race and Ethnicity**	
Black/African American	37.1
Hispanic/Latinx	30.4
White	16.5
Other	16.0
**Gender Identity**	
Cisgender Female	23.1
Cisgender Male	61.5
Transgender Female	4.1
Transgender Male	2.6
Non-binary/Gender Neutral/Other	8.7
**Sexual Orientation**	
Straight/Heterosexual	51.0
Gay	14.1
Lesbian	4.7
Bisexual	24.0
Other	6.3
**Age First Homeless**	
<16	21.1
16–17	24.2
18+	54.6
**Length of Most Recent Homelessness**	
A month or less	17.1
1–3 months	17.1
3 months – 1 year	31.6
1–2 years	12.4
>2 years	21.8
**Educational Experience**	
No High School Degree	32.0
High School Diploma/GED	42.3
More than High School Degree	25.8
**Currently Employed**	23.2
**Number of Days Drank 5 + Alcohol Drinks Last 30 Days**	
0	57.4
1–4	22.1
5+	20.5
**Number of Days Used Marijuana Last 30 Days**	
0	19.0
1–29	29.7
30 (every day)	51.3
**Number of Days Used Other Drugs Last 30 Days**	
0	63.1
1–4	15.9
5+	21.0
**% of Time Used Condoms With Primary Partners Last 3 Months**	
0% (never)	24.6
1–99% (sometimes)	6.3
100% (always)	69.1
**% of Time Used Condoms With Casual Partners Last 3 Months**	
0% (never)	9.8
1–99% (sometimes)	9.3
100% (always)	80.8
**Diagnosed with STI Last 3 Months**	7.2
**Been Tested for HIV Last 3 Months**	65.6
**Depressed Last 2 Weeks**	32.8
**Social Support from Friends Last 30 Days**	
Never / almost never	31.8
Sometimes	34.4
Almost always / always	33.9
**Any Mental Health Service Utilization Last 3 Months**	51.3
**Any Sexual Health Service Utilization Last 3 Months**	45.6
**Any Substance Use Service Utilization Last 3 Months**	38.0
**Any Housing Service Utilization Last 3 Months**	66.7
**Heard of PrEP (from 6-month survey)**	81.0
**Know Anyone Who has used PrEP (from 6-month survey)**	36.9
**Current or Past Use of PrEP (from 6-month survey)**	11.8
**Interested in PrEP (from 6-month survey)**	20.0
**Not Interested in PrEP (from 6-month survey)**	68.2

In terms of substance use and sexual behaviors, 42.6% reported heavy drinking behavior (5 or more drinks) during the last 30 days, 81% reported cannabis use during the last 30 days, and 36.9% reported using drugs other than cannabis in the last 30 days. In terms of sexual behavior, 30.9% reported condomless sex with primary partners in the last 3 months and 19.2% reported condomless sex with casual partners in the last 3 months. 7.2% reported having been diagnosed with an STI in the last 3 months and 65.6% reported having been tested for HIV in the last 3 months.

Regarding mental health and social support and service utilization, 32.8% of the sample screened positive for a probable depressive disorder during the last two weeks. In terms of social support, about one third (31.8%) reported never or almost never receiving social support, 34.4% reported sometimes receiving social support, and 33.9% reported always receiving social support when needed. Service utilization was quite high overall with 51.3% accessing mental health services, 45.6% accessing sexual health services, 38.0% accessing substance use services, and 66.7% accessing housing services in the last 3 months.

With respect to having awareness of PrEP, 158 (81.0%) YAEH reported that they had heard of PrEP, and 72 (36.9%) reported that they knew someone who had taken PrEP. With respect to the outcome for this analysis, interest and use of PrEP, YAEH were grouped into three categories based on: (a) current or prior use of PrEP; (b) no current/prior use but interest in PrEP; and (c) no current/prior use and no interest in PrEP. Twenty-three (11.8%) of the YAEH in this sample reported that they were currently using or had used PrEP in the past. An additional 39 (20.0%) YAEH in this sample reported that they were interested in taking PrEP. The remaining YAEH 133 (68.2%) reported that they had no interest in using PrEP.

### Bivariate Analysis

As a first step in our analysis, we assessed for bivariate associations between the individual, social and structural hypothesized to be associated with both PrEP interest and PrEP use (see Table 2).

**Table 2:** Characteristics of participants by use and interest in PrEP (N = 195)

Variable	Current/Past Use of PrEP (11.8%, n = 23)	Interested in PrEP (20.0%, n = 39)	Not Interested in PrEP (68.2%, n = 133)	Chi-square statistic (df)
**Age**				3.8 (2)
18–20	5.3	13.2	81.6	
21–25	13.4	21.7	65.0	
**Race and Ethnicity**				5.1 (6)
Black/African American	11.1	13.9	75.0	
Hispanic/Latinx	11.9	27.1	61.0	
White	15.6	15.6	68.8	
Other	9.7	25.8	64.5	
**Sexual and Gender Minority Status**				20.0 (4) ***
Cisgender Females/Straight Cisgender Males	4.1	17.4	78.5	
Gay and Bisexual Cisgender Males	22.7	29.6	47.7	
Gender Diverse Persons	26.7	16.7	56.7	
**Age First Homeless**				3.8 (2)
< 16	4.9	22.0	73.2	
16–17	8.5	19.2	72.3	
18+	16.0	19.8	64.2	
**Length of Recent Homelessness**				4.3 (2)
A month or less	15.2	21.2	63.6	
1–3 months	18.2	27.3	54.6	
3 months – 1 year	11.5	21.3	67.2	
1–2 years	8.3	16.7	75.0	
> 2 years	7.1	14.3	78.6	
**Educational Experience**				3.5 (2)
No High School Degree	6.5	19.4	74.2	
High School Diploma/GED	12.2	22.0	65.9	
More than High School Degree	18.0	18.0	64.0	
**Employment**				2.8 (2)
Currently employed	11.1	28.9	60.0	
Unemployed	11.4	17.5	71.1	
**Number of Days Heavy Drinking**				6.0 (2)+
0	16.1	20.5	63.4	
1–4	9.3	23.3	67.4	
5+	2.5	15.0	82.5	
**Number of Days Used Marijuana**				2.3 (2)
0	21.6	18.9	59.5	
1–29	13.8	25.9	60.3	
30 (every day)	7.0	17.0	76.0	
**Number of Days Used Other Drugs**				4.5 (2)
0	16.3	19.5	64.2	
1–4	3.2	19.4	77.4	
5+	4.9	22.0	73.2	
**% Condom Use, Primary Partners**				2.0 (2)
0% (never)	8.5	14.9	76.6	
1–99% (sometimes)	8.3	25.0	66.7	
100% (always)	12.9	22.0	65.2	
**% Condoms Use, Casual Partners**				4.5 (2)
0% (never)	5.3	36.8	57.9	
1–99% (sometimes)	16.7	27.8	55.6	
100% (always)	12.2	17.3	70.5	
**Diagnosed with STI**				2.5 (2)
No	11.6	18.8	69.6	
Yes	14.3	35.7	50.0	
**Been Tested for HIV**				3.1 (2)
No	16.4	14.9	68.7	
Yes	9.4	22.7	68.0	
**Depression**				4.5 (2)
No	13.7	16.0	70.2	
Yes	7.8	28.1	64.1	
**Social Support from Friends**				0.5 (2)
Never / almost never	14.5	17.7	67.7	
Sometimes	7.5	19.4	73.1	
Almost always / always	13.6	22.7	63.6	
**Mental Health Service Utilization**				1.8 (2)
No	14.9	18.1	67.0	
Yes	9.1	22.2	68.7	
**Sexual Health Service Utilization**				4.9 (2)+
No	12.3	14.2	73.6	
Yes	11.2	27.0	61.8	
**Substance Use Service Utilization**				0.7 (2)
No	13.2	19.0	67.8	
Yes	9.5	21.6	68.9	
**Housing Service Utilization**				2.6 (2)
No	16.9	16.9	66.2	
Yes	9.2	21.5	69.2	
**Heard of PrEP**				2.1 (2)
No	8.1	13.5	78.4	
Yes	12.7	21.5	65.8	
**Know anyone who has used PrEP**				25.6(2) ***
No	2.4	16.3	81.3	
Yes	27.8	26.4	45.8	

*Note*. + *p* < 0.10. *** *p* < 0.001 for association between PrEP use or interest and participant characteristic per chi-square test from a multinomial logistic regression modeling the three-category outcome with the characteristic

#### Individual Factors

Sexual and gender minority status significantly predicted both current/past PrEP use and current interest in using PrEP. Specifically, gay and bisexual cisgender identified men were more likely than cisgender women or cisgender straight identified men to report current or past PrEP use than to report not being interested in using PrEP [odds ratio (*OR*) = 9.05, 95% confidence interval (*CI*)=(2.80, 29.24), *p* < 0.001]. Gay and bisexual cisgender identified men were also more likely than cisgender women or cisgender straight identified men to report being interested in using PrEP than to report not being interested in using PrEP [*OR* = 2.80, 95% *CI*=(1.21, 6.47), *p* < 0.05]. Gender diverse individuals were more likely than cisgender women or cisgender straight identified men to report current or past PrEP use than to report not being interested in using PrEP [*OR* = 8.91, 95% *CI*=(2.61, 30.61), *p* < 0.001].

For substance use, participants who reported more days of heavy alcohol use in the last 30 days were less likely to report current or past use of PrEP than to report not being interested in using PrEP [*OR* = 0.40, 95% *CI*= (0.19, 0.87), *p* < 0.05].

#### Social Factors

Participants who reported knowing someone who had used PrEP were more likely to report past or current use of PrEP themselves (*n* = 20, 27.8%) than to report not being interested in using PrEP [*OR* = 20.20, 95% *CI=*(5.60, 72.33), *p* < 0.001]. Participants who reported knowing someone who had used PrEP were also more likely to report being interested in using PrEP (*n* = 19, 26.4%) than to report not being interested in using PrEP [*OR* = 2.88, 95% *CI=*(1.37, 6.04), *p* = 0.01].

#### Structural Factors

Finally, examination of structural factors indicated that participants who recently accessed sexual health services were marginally more likely to report interest in PrEP (*n* = 24, 27.0%) than were those who did not recently access sexual health services (*n* = 15, 14.2%) [*OR* = 2.27, 95% *CI=*(1.09, 4.72), *p* < 0.05].

### Multivariable Models

Each of the variables found to have at least a marginal (*p* < 0.10) association with the three-level PrEP use and interest outcome variable (current/past PrEP use, interested in PrEP, or no interest in PrEP) was then entered into a multivariable model. In the bivariate multinomial models described above, sexual and gender minority status, last-month heavy drinking (days had 5 + drinks), sexual health service utilization during the last 3 months, and knowing someone who has used PrEP were all found to have at least marginal associations and were thus entered into the multivariable model.

Table 3 illustrates effects of the bivariately significant or marginal predictor variables together in one model on the probabilities of reporting current or past PrEP use and of being interested in using PrEP vs. being not using or interested in using PrEP.

**Table 3:** Multivariable multinomial logistic regression results modeling PrEP use and PrEP interest

	Adjusted Odds Ratio (95% Confidence Interval), *p*-value	
Variable	Current/Past PrEP Use vs. No Interest in PrEP	Interested in PrEP vs. No Interest in PrEP
Gay and Bisexual Cisgender Males (vs. Cisgender Females/Straight Cisgender Males)	5.25 (1.38, 19.93), *p* = 0.02	2.22 (0.87, 5.70), *p* = 0.10
Gender Diverse Persons (vs. Cisgender Females/Straight Cisgender Males)	5.21 (1.41, 19.32), *p* = 0.01	0.94 (0.29, 3.08), *p* = 0.92
Number of Days Drank 5 + Alcohol Drinks, Last 30 Days ^a^	0.36 (0.16, 0.86), *p* = 0.02	0.76 (0.47, 1.22), *p* = 0.25
Any Sexual Health Service Utilization, Last 3 Months	0.94 (0.33, 2.63), *p* = 0.90	2.22 (1.03, 4.76), *p* < 0.05
Know Someone Who Has Used PrEP	5.29 (1.69, 16.57), *p* = 0.004	2.35 (1.02, 5.39), *p* < 0.05

*Note*. p-value for association between PrEP use or interest and participant characteristic, adjusted for all other characteristics, per chi-square test from a multinomial logistic regression modeling the three-level outcome with all four characteristics

^a^ Tested as displayed in Tables 1 and 2, with ordinal categories 0 days (1), 1–4 days (2), and 5 + days (3)

When comparing those YAEH who endorsed current or past PrEP use to those who were not interested, results showed that those who identified as gay or bisexual cisgender men were significantly more likely to endorse current or past use of PrEP as compared to cisgender women or straight identified cisgender men [*OR* = 5.25, 95% *CI*=(1.38, 19.93), *p* < 0.05]. In addition, those who identified as gender diverse were significantly more likely to endorse current or past use of PrEP as compared to cisgender women or straight identified cisgender men [*OR* = 5.21, 95% *CI*=(1.41, 19.32), *p* = .05]. The model suggests that those who reported more days of heavy drinking in the last 30 days were significantly less likely to endorse current or past use of PrEP [*OR* = 0.36, 95% *CI*=(0.16, 0.86), *p* = .05]. Finally, the model suggests that that those who reported knowing someone who has used PrEP were significantly more likely to endorse current or past use of PrEP compared to those who reported not knowing someone who has used PrEP [*OR* = 5.29, 95% *CI*=(1.69, 16.27), *p* = .01].

#### Interest in PrEP vs. Not Interested in PrEP

When comparing those YAEH who endorsed current interest in PrEP to those who did not, the model suggests that those who recently accessed sexual health services were significantly more likely to be interested in using PrEP [*OR* = 2.22, 95% *CI*=(1.03, 4.76), *p* = 0.04]. In addition, the model suggests that those who knew someone who had used PrEP were significantly more likely to be interested in using PrEP [*OR* = 2.35, 95% *CI*=(1.02, 5.39), *p* = 0.04].

## Discussion

YAEH have disproportionately high risk for HIV compared to their stably housed peers [24]. This study sought to assess predictors of PrEP use and interest among a racially, ethnically, and SGM diverse sample of YAEH who report one or more behavioral risk factors associated with HIV. Prior work suggests that although awareness of PrEP has been relatively low among YAEH, many report willingness to take PrEP once informed about it [25]. Among those YAEH found to meet risk criteria for HIV in our current Los Angeles-based sample, over 80% reported that they were aware of PrEP and more than 1 in 3 reported knowing someone who had taken PrEP. However, about two-thirds reported that they were not interested in taking PrEP, with rates of interest in taking PrEP (20%) and use of PrEP in the past (11.8%) being relatively low.

We aimed to elucidate key individual, social, and structural factors associated with interest and use of PrEP among YAEH at risk for HIV. At the individual level, identifying as either a cisgender sexual minority male or as a person who is gender diverse was significantly associated with PrEP interest among YAEH. This is not surprising given that outreach and marketing for PrEP interventions has specifically focused on sexual and gender minority individuals [40, 41]. Given the disproportionate risk for HIV, it seems appropriate that there would be more interest among YAEH who identify as members of certain sexual and gender minority groups as they may be at higher relative risk. It is important to recognize that all YAEH participants from all sexual and gender categories met criteria for at least one known HIV risk factor. Therefore, it is important that future PrEP interventions targeting YAEH include members of all sexualities and gender identities in their outreach and marketing materials and ensure that intervention content is culturally appropriate and representative of all YAEH who may be at risk.

More frequent heavy drinking was associated with a lower likelihood of current or past use of PrEP. This finding highlights the importance of integrating PrEP into existing behavioral health interventions that simultaneously address mental health, substance use, and sexual health needs of YAEH. Long-acting injectable formulations of PrEP may be a particularly useful intervention for YAEH with mental health and substance use concerns that may affect PrEP adherence and persistence.

On the social level, peer influence may be a particularly salient motivator for PrEP use. Knowing someone who had taken PrEP was a strong correlate of both interest in and use of PrEP among YAEH. According to social norms theory, individuals often overestimate the number of their peers who engage in “risk” behaviors and underestimate the number of their peers who engage in “protective” behaviors [42]. These misperceptions may cause individuals to act in a way that is consistent with the misperceived social norm [42]. In the example of engaging in a health behavior like PrEP, YAEH may be less likely to take PrEP if they do not believe anyone in their peer group is taking it. PrEP-related stigma has also been reported as a significant barrier to PrEP use among YAEH [23, 43]. Knowing someone who has taken or is currently taking PrEP may be an indication that PrEP use has been normalized among members one’s peer group. Peers or other members of their social network may serve as role models providing examples of who should take PrEP and why it is important. Future work to increase uptake of PrEP among YAEH should focus on reducing misperceptions about PrEP use and PrEP-related stigma within the social networks of YAEH by encouraging open discussions about PrEP use with their peers. A promising social network-based PrEP interventions could be adapted for use among YAEH [44–47].

Finally, on a structural level, it seems key that information about PrEP and linkage to PrEP programs take place in the context of HIV and STI counseling and health service programs. Our results indicate that YAEH who recently received these services appear to be particularly interested in PrEP and are likely to be good candidates for PrEP use. Offering long-acting injectable PrEP at the same time YAEH are receiving other HIV/STI health services may be a particularly useful way of capitalizing on their interest in PrEP. While offering PrEP or information about PrEP to YAEH in sexual health service setting may seem intuitive, many providers hesitate to offer PrEP to youth under 18 because of their perceptions of potential ethical and legal issues when prescribing PrEP to a minor [31].

This study was not without limitations. For example, the study was limited to young adults recruited from the Los Angeles area. PrEP information and medication is possibly more accessible to YAEH in the Los Angeles metropolitan area than in some other areas of the country due to state-funded PrEP coverage programs and multiple ongoing interventions focused on increasing knowledge and access to PrEP among youth in the Los Angeles area. As a result, results from this study may not generalize to YAEH in other areas of the country. This study was also limited to self-report data and, as such, we are unable to confirm whether those who reported PrEP use were indeed taking PrEP. Finally, as PrEP was not the original focus of the study, we are limited in the number and type of PrEP-related questions assessed. Future work should extend PrEP questions to focus on how and why participants may be interested or not interested in using PrEP and on preferences for PrEP formulation (e.g. long-acting, daily pills) and delivery (e.g., nurse administered in drop-in centers, pharmacist delivered in community pharmacies).

This study extends prior work suggesting that YAEH have a general interest in taking PrEP once they are aware of it, [23, 25] by finding that YAEH who identify as sexual and/or gender minority, know someone who had used PrEP, or recently accessed sexual health services are more likely to have used and/or report interest in using PrEP. Those who reported more episodes of heavy drinking were less likely to report having used PrEP. Our study suggests that interventions focused on increasing information and uptake of PrEP may be well received in drop-in centers offering mental and sexual health care services to YAEH. Finally, leveraging peer groups of YAEH and fostering positive social norms around PrEP use are likely to help further reduce PrEP-related stigma and create a sense of normality around taking PrEP. Given the relatively high risk for HIV among YAEH, the high efficacy of PrEP, and the recent approval of a long-acting injectable formulation, efforts must focus on increasing access and uptake of PrEP among YAEH to end the United States HIV epidemic.
